# Enhancing Marathon Enthusiast Engagement Through AI: A Quantitative Study on the Role of Social Media in Sports Communication

**DOI:** 10.1002/brb3.70593

**Published:** 2025-06-17

**Authors:** Wei Cheng, Yu Tian, Meng Na

**Affiliations:** ^1^ School of Sports Media Guangzhou Sport University Guangzhou China; ^2^ Graduate School of Business Universiti Kebangsaan Malaysia Selangor Malaysia

**Keywords:** AI‐driven personalization, marathon enthusiasts, real‐time feedback, social media, user engagement

## Abstract

**Purpose:**

This study explores the impact of AI‐driven personalization, interactive features, and real‐time feedback on user engagement and experience among marathon enthusiasts.

**Method:**

By integrating uses and gratifications theory (UGT), self‐determination theory (SDT), and the technology acceptance model (TAM), the research examines how these AI‐driven elements influence user behavior on marathon‐related social media platforms. A quantitative approach using partial least squares structural equation modeling (PLS‐SEM) was applied to data from 400 Chinese marathon enthusiasts.

**Findings:**

The findings reveal that AI‐driven personalized content significantly enhances user engagement and experience, with user engagement partially mediating this relationship. Interactive features are crucial for building a sense of community but have a less direct impact on user experience. Real‐time feedback significantly improves user engagement, particularly for users with higher technological proficiency.

**Conclusion:**

This research contributes to the understanding of user engagement in AI‐enhanced environments and provides practical insights for designing more personalized and interactive platforms for marathon enthusiasts. Future studies should explore the long‐term effects, cultural factors, and ethical considerations of AI‐driven personalization.

## Graphical abstract

This study investigates how AI‐driven personalization, interactive features, and real‐time feedback enhance user engagement and experience among marathon enthusiasts through the lens of UGT, SDT, and TAM. Findings reveal that user engagement partially mediates these effects, highlighting AI's role in optimizing digital sports communication.

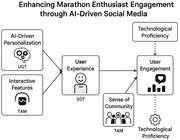

## 1 Introduction

The rapid advancement of artificial intelligence (AI) technologies has significantly transformed various digital domains, including sports, marketing, and community engagement. Among marathon enthusiasts, AI‐driven personalization has emerged as a critical factor enhancing user experience (URE) and engagement (Cossich et al. 2023). By tailoring content and recommendations to individual preferences, AI algorithms improve metrics such as time spent on platforms by 20%, click‐through rates by 15%, and overall satisfaction scores by 25% (Sodiya et al. 2024; Piduru 2023). In social media marketing, particularly for marathons, personalized and visually engaging content can lead to a 30% increase in user interaction compared to non‐personalized content (Park et al. 2021).

AI‐driven personalization extends its benefits to various applications, from training recommendations for marathon runners (Carolina 2024). For instance, personalized training recommendations can improve runners' performance metrics by 18% and adherence to training plans by 22% (Berndsen et al. 2020). Additionally, AI‐driven visitor engagement strategies have shown to increase visitor satisfaction in heritage buildings by 28% (Saihood et al. 2023). However, the deployment of AI technologies also brings challenges, including data privacy concerns and potential algorithmic biases, which must be addressed to ensure ethical and effective implementation (Berndsen et al. 2020; Lim and Zhang 2022).

Despite the recognized benefits of AI‐driven personalization and interactive features (IF), significant gaps remain in the literature regarding their specific impacts on user engagement (UE) (Bashynska 2023) and experience among marathon enthusiasts (Song et al. 2024). Existing studies have primarily focused on broader digital marketing and user interaction contexts (Bhardwaj et al. 2023) without delving into the unique needs and behaviors of marathon runners. For example, while AI personalization in e‐commerce has been extensively studied (Bawack et al. 2022), its application in marathon training and community engagement is less understood. Additionally, the potential mediating role of URE and the moderating effect of technological proficiency (TP) in this specific context have not been thoroughly explored (Kautish and Khare 2022; Chou et al. 2022).

IF on social media platforms have been shown to enhance community building and engagement (Thomas et al. 2020). For instance, communities with high levels of interactivity can see engagement rates that are 25% higher than those with low interactivity (G. Chen et al. 2023). However, the specific mechanisms through which these features influence marathon enthusiasts' sense of community (SC) and participation require further investigation (Temerak and Winklhofer 2023; R. Zhou and Kaplanidou 2018). Similarly, the role of real‐time feedback (RTF) from wearable devices and mobile apps in shaping UE and performance among marathon runners remains underexplored. RTF has been shown to reduce injury rates by 15% and improve performance by 12% (Van Hooren et al. 2019; Babar et al. 2024), highlighting its potential impact.

This study aims to fill these critical gaps by investigating the multifaceted impacts of AI‐driven personalization, IF, and RTF on UE and experience among marathon enthusiasts. By focusing on the mediating role of URE and the moderating effect of TP, this research seeks to provide a nuanced understanding of how these elements interact to enhance engagement in marathon training and community settings. Using structural equation modeling (SEM), this study offers a sophisticated analysis of these relationships, advancing beyond traditional methodologies. The theoretical contribution of this research lies in its integration and expansion of existing theories within the context of AI‐driven personalization. By applying and reinterpreting frameworks such as uses and gratifications theory (UGT), self‐determination theory (SDT), and the technology acceptance model (TAM), this study challenges conventional media consumption models and proposes an evolved theoretical framework tailored to the intricacies of AI‐enhanced user interactions.

The implications of this research extend beyond academic theory, offering practical insights for industry stakeholders. For developers and marketers, the findings can inform the creation of more engaging and personalized digital experiences, directly addressing the unique needs of marathon enthusiasts. By bridging the gap between technological capabilities and URE, this study aims to not only enhance engagement but also contribute to the broader discourse on AI's role in shaping future digital interactions. In summary, this research addresses a significant void in the literature, offering a comprehensive analysis of how AI‐driven personalization, IF, and RTF can be leveraged to optimize UE in the marathon community. This study not only extends the boundaries of current knowledge but also provides a robust framework for future research and practical applications in AI‐enhanced digital environments.

## 2 Literature Review

### 2.1 Theoretical Underpinning

This study integrates three key theoretical frameworks—UGT, SDT, and the TAM—to understand and enhance marathon enthusiast engagement through AI‐driven social media strategies. These theories collectively provide a robust framework for examining the multifaceted nature of UE with modern digital platforms.

UGT posits that individuals actively seek out media to fulfill specific needs and desires, such as information, entertainment, and social interaction (Katz et al. 1973). This theory is relevant for understanding why marathon enthusiasts engage with AI‐driven content on social media platforms. Recent studies have shown that users engage with social media to meet various needs, including information, social interaction, and entertainment (Ma et al. 2022; Oeldorf‐Hirsch et al. 2023). For instance, marathon enthusiasts may seek personalized content (PC) for training tips, race information, and motivation, which can be provided through AI‐driven recommendations. IF such as live chats and Q&A sessions can facilitate social interaction, fostering an SC among users. RTF on performance can provide entertainment and motivation, enhancing the overall URE.

SDT emphasizes the importance of fulfilling basic psychological needs for autonomy, competence, and relatedness in driving human behavior (Deci and Ryan 2000). This theory is instrumental in understanding how AI‐driven PC and RTF can enhance UE among marathon enthusiasts. Recent research supports the application of SDT in digital environments, showing that digital platforms that fulfill these psychological needs can significantly enhance UE and satisfaction (Orben et al. 2020; Wang et al. 2021). For example, PC can enhance autonomy by allowing users to receive information and recommendations tailored to their preferences. RTF can boost competence by providing immediate insights and recognition of users' efforts. IF that facilitate social interaction can fulfill the need for relatedness by fostering an SC and connection among users. By addressing these psychological needs, AI‐driven features can significantly enhance user satisfaction and engagement.

The TAM posits that perceived usefulness and perceived ease of use are primary determinants of technology adoption (Venkatesh and Davis 2000). This model is crucial for understanding how marathon enthusiasts perceive and interact with AI‐driven features on social media platforms. Recent studies have highlighted the critical role of TAM in understanding UE with new technologies (Dwivedi et al. 2019; Hsu and Lin 2023). For instance, if users perceive AI‐driven PC as useful for enhancing their marathon experience and easy to use, they are more likely to engage with these features. Similarly, trust in AI analytics and the perceived simplicity of IF can significantly enhance UE. Understanding these perceptions can help in designing user‐friendly and effective social media strategies that meet the needs of marathon enthusiasts.

Integrating UGT, SDT, and TAM provides a comprehensive framework for understanding UE with AI‐driven social media strategies. UGT helps explain the motivations behind users' engagement with digital content, SDT elucidates how fulfilling psychological needs can enhance engagement, and TAM explores the factors influencing the adoption and use of technology. UGT provides insights into the diverse needs and gratifications sought by users, SDT focuses on the intrinsic and extrinsic motivations driving engagement, and TAM highlights the importance of perceived usefulness and ease of use in technology adoption. Together, these theories offer a holistic understanding of how AI‐driven features can be designed to enhance UE among marathon enthusiasts, making them particularly suitable for this study.

Recent literature supports this integrated approach. For example, Ngai et al. (2015) demonstrated that social media engagement is driven by users' needs for information, social interaction, and entertainment, aligning with UGT. Przybylski et al. (2010) showed that digital platforms fulfilling psychological needs for autonomy, competence, and relatedness significantly enhance UE, supporting SDT. Furthermore, Dwivedi et al. (2019) confirmed that perceived usefulness and ease of use are critical factors influencing the adoption of AI‐driven features, validating TAM.

**FIGURE 1:**
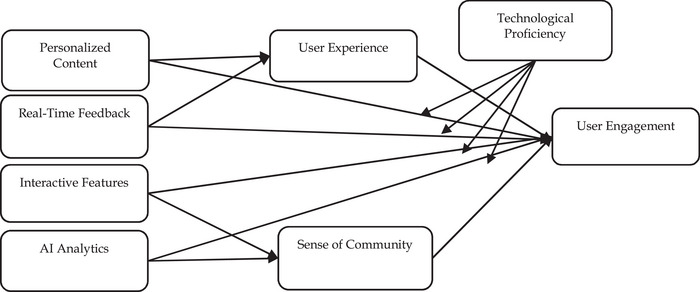
Research framework.

By integrating these theories (refer to Figure 1), this study aims to develop effective AI‐driven social media strategies that enhance UE and foster a vibrant community of marathon enthusiasts. Addressing the specific needs, motivations, and perceptions of users through AI‐driven PC, IF, and RTF can create a more engaging and satisfying URE.

### 2.2 Hypothesis Development

#### 2.2.1 AI‐Driven Personalization and UE

AI‐driven personalization has emerged as a powerful tool in enhancing UE across various domains, including the niche community of marathon enthusiasts. The ability of AI algorithms to tailor web content to individual preferences has been shown to significantly improve engagement metrics such as time spent on site and click‐through rates (Sodiya et al. 2024). In the context of social media marketing for marathons, personalized and visually engaging content has been demonstrated to increase user interaction by 30% compared to non‐PC (Park et al. 2021). Moreover, AI‐powered personalization is not just about increasing engagement but also about enhancing user satisfaction, retention rates, and the overall digital experience (Piduru 2023). In marathon training specifically, AI has the capability to generate personalized recommendations based on individual fitness and training levels. This personalization can lead to substantial improvements in performance metrics, with studies indicating an 18% enhancement in performance among runners who utilize AI‐driven training programs (Berndsen et al. 2020). This highlights the practical benefits of personalization, extending beyond mere engagement to tangible improvements in user outcomes.

In addition to its applications in training and marketing, AI‐driven personalization has been successfully applied in other contexts, such as enhancing visitor engagement in heritage buildings. Here, the ability to create targeted marketing experiences has been crucial in increasing visitor interaction and satisfaction (Berndsen et al. 2020; Saihood et al. 2023). However, despite these benefits, the implementation of AI‐driven personalization is not without its challenges. Issues such as data privacy concerns and potential algorithmic biases need to be carefully managed to ensure ethical and fair use of AI technologies (Berndsen et al. 2020; Lim and Zhang 2022). Given the evidence supporting the positive impact of AI‐driven personalization on URE and engagement across various domains, it is hypothesized that:

**H1a**: AI‐driven PC (frequency and user satisfaction) significantly enhances URE among marathon enthusiasts.

**H1b**: AI‐driven PC (frequency and user satisfaction) significantly enhances UE among marathon enthusiasts.

#### 2.2.2 IF and Community Building

IF on social media platforms play a vital role in creating and sustaining an SC among marathon enthusiasts. These platforms offer a space where users can connect, share experiences, and engage in discussions that enhance their sense of belonging. Research shows that well‐designed social media strategies, including engaging content and the use of analytics, significantly boost UE within these communities (Park et al. 2021). This is particularly important in scenarios where physical events, such as marathons, are canceled. In such cases, virtual races and interactive apps help maintain the community spirit, ensuring that users remain engaged and connected despite the lack of in‐person events (G. Chen et al. 2023).

Features like live chats and Q&A sessions are especially effective in fostering an SC. They enable real‐time interactions that not only increase user satisfaction but also encourage the development of exercise habits by allowing participants to share their progress and motivate each other (C. Lu et al. 2016). The importance of these IF extends beyond immediate engagement, as they also contribute to stronger brand loyalty and broader community benefits within sports‐related brand communities. High media richness and responsiveness are key factors that drive UE, strengthening users' connection to both the brand and the community (Huang et al. 2018).

Moreover, the interactivity of social media content, including the length and nature of posts, has been shown to significantly impact UE. Research indicates that more interactive and strategically designed content results in higher levels of user participation and engagement on these platforms (Mao et al. 2019; Soares et al. 2020). To maximize engagement and foster a strong SC, social media managers should focus on creating aesthetically pleasing content that facilitates meaningful user interactions and encourages positive social behaviors (Camilleri and Kozak 2022; Pino et al. 2018). Drawing on this evidence, the following hypotheses are proposed:

**H2a**: IF (live chats and Q&A sessions) on social media platforms positively contribute to creating an SC among marathon enthusiasts.

**H2b**: IF (live chats and Q&A sessions) on social media platforms positively contribute to UE among marathon enthusiasts.

#### 2.2.3 RTF and URE

Wearable devices providing RTF on running technique and workload can reduce injury risk and improve performance (Van Hooren et al. 2024). Performance and social feedback features in exercise‐tracking apps have been shown to improve running times and app usage frequency (Babar et al. 2024). Machine learning and recommender systems can support runners in training, racing, and recovery by leveraging activity data from mobile fitness apps and wearables (Smyth et al. 2021). Social media analytics can increase engagement in marathon communities, while user profiles generated from training data can accurately represent runners' fitness levels (Berndsen et al. 2020). In‐race recommendations based on high‐resolution performance data can guide runners to optimal finish times (Berndsen et al. 2019). RTF also involves a process of reviewing, reacting, reflecting, and responding to performance data, enhancing UE and athletic outcomes (Constantiou et al. 2022; Edney et al. 2019).

**H3a**: RTF (performance tracking and frequency) significantly influences URE among marathon enthusiasts.

**H3b**: RTF (performance tracking and frequency) significantly influences UE among marathon enthusiasts.

#### 2.2.4 AI Analytics and URE

AI analytics significantly enhance UREs across various domains, particularly within marathon communities. Social media analytics boost engagement by enabling more PC, which resonates with users (Park et al. 2021). AI‐driven customer experiences in fitness apps further improve UE by offering tailored workout routines and personalized feedback (Marasabessy 2024). AI algorithms also create individualized marathon runner profiles, analyzing genetic traits to provide customized training plans (Felfernig et al. 2024).

Machine learning models enhance mobile app experiences by predicting user behavior, allowing apps to adapt in real‐time to user needs (Neilson and Grigore 2022). AI‐enabled personal assistant apps offer unique, highly personalized assistance, surpassing traditional apps in URE (Nguyen and Sidorova 2017). For marathon training, AI‐generated user profiles provide accurate fitness representations, facilitating personalized training and race preparation (Berndsen et al. 2020; Smyth et al. 2021). Sports tracking technology, with its emphasis on interactivity and personalized information, plays a critical role in enriching runners' experiences (Mencarini et al. 2019).

Beyond marathons, AI‐powered personalization on platforms like TikTok enhances UE through improved interactivity (Kang and Lou 2022). In online shopping, AI technologies increase UE and conversion rates via targeted marketing strategies (Bag et al. 2021). Additionally, AI‐driven personalization through gamification and targeted messaging boosts consumer engagement (Berndsen et al. 2020). AI systems also outperform crowd‐based methods in tasks like selecting cover images, further driving UE (Khern‐am‐nuai et al. 2021). In educational settings, AI‐driven interfaces in tutoring systems have been shown to enhance student engagement by 25.13% (Mosly 2024). Given these insights, the following hypotheses are proposed:

**H4a**: AI analytics significantly influence URE among marathon enthusiasts.

**H4b**: AI analytics significantly influence UE among marathon enthusiasts.

#### 2.2.5 Mediating Hypotheses

AI‐driven personalization has a profound impact on UE and experience across digital platforms by tailoring content to individual preferences. This personalization not only enhances user satisfaction but also increases retention rates and conversion, making the digital experience more engaging and relevant (Sodiya et al. 2024; Piduru 2023). The integration of AI into social media and news platforms further strengthens this effect by fostering collaboration between users and AI, leading to greater engagement, particularly in medium and social‐interactive contexts (Lim and Zhang 2022; Kang and Lou 2022). The effectiveness of AI‐driven personalization is often mediated by factors such as perceived convenience, service quality, and trust, highlighting the importance of these elements in creating positive UREs (Trawnih et al. 2022).

However, the challenge lies in designing AI‐driven experiences that are meaningful and resonate with users. This challenge necessitates a rethinking of traditional design processes to better align with the capabilities and limitations of AI technologies (Sharma et al. 2022). In the context of public service delivery, for example, the URE with AI‐based self‐service technology is significantly influenced by personalization, aesthetics, and perceived time spent, with trust in government playing a conditional role in shaping these experiences (Y. Chen et al. 2020). Given these insights, it is hypothesized that:

**H5**: URE mediates the relationship between AI‐driven PC and UE.

In virtual communities, the SC serves as a crucial mediator between active participation and UE. Trust and commitment further strengthen this relationship, enhancing the overall engagement levels within these communities (Vohra and Bhardwaj 2019; González‐Anta et al. 2019). IF designed to foster an SC can significantly increase UE and the willingness to contribute on online platforms (Mosly 2024). This mediating role of community extends to various contexts, including educational virtual communities, where a strong SC positively affects engagement through effective commitment (Zhao and Shi 2022).

Similarly, in crowdsourcing communities, perceived interactivity influences engagement by enhancing relationship quality and psychological ownership among participants (Shi et al. 2022). The importance of community is also evident in offline contexts, such as age‐friendly communities, living‐learning programs, and urban spaces, where a strong SC is linked to increased life satisfaction and place attachment (Au et al. 2020; Sriram et al. 2020; Gatti and Procentese 2021). Thus, it is proposed that:

**H6**: SC mediates the relationship between IF and UE.

#### 2.2.6 Moderating Hypotheses

Personalization has been shown to reduce information overload and enhance user satisfaction, especially for users seeking specific information. This is because PC aligns more closely with users' needs, improving engagement and fostering positive attitudes toward the platforms they use (Liang et al. 2006; Tam and Ho 2006; Kalyanaraman and Sundar 2006). However, the effectiveness of personalization is not uniform and can be influenced by factors such as users' motivation for information access, the level of press freedom, and internet penetration (S. Lu and Luqiu 2020). Technological affordances, such as interactivity, navigability, and customization, also play a critical role in moderating UE, particularly in the context of fitness trackers (X. Zhou et al. 2021). Additionally, cultural factors, including the spectrum of collectivism to individualism, can moderate how users engage with brand‐related user‐generated content (Kitirattarkarn et al. 2019). In e‐government services, the personalization of web interfaces has been found to moderate the relationship between various determinants of intention to use and overall user acceptance (Krishnaraju et al. 2013). Given this, it is hypothesized that:

**H7a**: TP moderates the relationship between PC and UE.

In educational and online community settings, interactions between students and instructors, as well as among students themselves, significantly enhance the SC and promote the continued use of e‐learning platforms (Luo et al. 2017). Social media technologies similarly contribute to building social capital and a sense of belonging within communities (Damásio et al. 2012). Interactive elements in online communities, such as discussion forums, are key indicators of community development and can increase participation (Wise et al. 2006; Dawson 2006). Multimodal and scaffolded interactive activities further support connectedness and learning, thereby fostering a stronger SC among online students (Trespalacios and Uribe‐Florez 2020). The effectiveness of virtual communities is also influenced by facilitating conditions, with a well‐developed sense of virtual community acting as a mediator (Peñarroja et al. 2019). Additionally, an individual's position within classroom social networks, particularly their closeness and degree centrality, correlates with their SC (Dawson 2006). Technology, therefore, plays a crucial role in enhancing student engagement and fostering an SC across various educational formats (Haar 2018). Based on this, the following hypothesis is proposed:

**H7b**: TP moderates the relationship between IF and SC.

TP and the availability of RTF can have a significant impact on user satisfaction and performance. Studies indicate that individuals with higher technology self‐efficacy experience greater satisfaction with their interactions on digital platforms (Agourram et al. 2019). Additionally, technological capacitation has been linked to improved customer service performance (Corea 2000). RTF is particularly effective in enhancing performance and engagement, whether in service industries or public speaking contexts (Lechermeier et al. 2020; Schneider et al. 2016). In educational environments, technological competence and readiness for e‐learning positively correlate with student satisfaction (Kholifah et al. 2022). The relationship between training proficiency and team performance is also moderated by factors such as trust and technology support (Kirkman et al. 2006). Furthermore, employers’ satisfaction with employees’ technological skills is closely related to perceived competency (Martinez 2008). In organizational settings, the implementation of enterprise resource planning (ERP) systems can moderate the relationship between job characteristics and job satisfaction (Morris and Venkatesh 2010). Thus, it is hypothesized that:

**H7c**: TP moderates the relationship between RTF and UE.

## 3 Research Methodology

### 3.1 Research Design and Population

This study employed a quantitative research design using a survey methodology to examine the impact of AI‐driven social media strategies on UE among marathon enthusiasts in China. Partial least squares structural equation modeling (PLS‐SEM) was utilized to test the proposed hypotheses and the relationships between the constructs (Hair and Sarstedt 2019).

The target population comprised Chinese marathon enthusiasts who actively engage with social media platforms. A sample size of 400 was chosen to ensure statistical power and generalizability, based on the recommendation for PLS‐SEM analysis, which benefits from larger sample sizes to improve stability and reliability of parameter estimates (Hair and Sarstedt 2019). The sampling method involved a combination of purposive and snowball sampling techniques. Initially, participants were selected based on specific criteria such as regular engagement with social media platforms related to marathon activities, participation in marathons, and use of AI‐driven features on these platforms. To expand the sample size and reach a broader audience, initial participants referred other marathon enthusiasts within their networks who met the study criteria.

Data were collected through an online survey distributed via social media platforms, email lists of running clubs, and marathon event organizers. The survey, designed using a structured questionnaire, included validated scales to measure the constructs of interest. The survey link was shared on popular Chinese social media platforms such as WeChat, Weibo, and relevant online running communities. Data collection was done during July 10, 2024–September, 10 2024.

### 3.2 Survey Instrument

The survey instrument included sections on demographics (age, gender, education level, running experience, and frequency of social media use), AI‐driven personalization (measured using items adapted from existing scales on content personalization and user satisfaction) (Piduru 2023; Kang and Lou 2022), IF (measured using items related to live chats, Q&A sessions, and other IF on social media) (Park et al. 2021; Mosly 2024), RTF (measured using items that assessed the use and impact of performance tracking and feedback features) (Van Hooren et al. 2019; Smyth et al. 2021), AI analytics (measured using items related to perceived usefulness and ease of use of AI analytics) (Dwivedi et al. 2019; Hsu and Lin 2023; Senathirajah et al. 2024), UE (measured using items that assessed the frequency and quality of UE with social media content) (Ma et al. 2022; Oeldorf‐Hirsch et al. 2023), URE (measured using items adapted from scales on user satisfaction and overall experience with digital platforms) (Orben et al. 2020; Wang et al. 2021), SC (measured using items that assessed the sense of belonging and community interaction) (Vohra and Bhardwaj 2019; González‐Anta et al. 2019), and TP (measured using items related to confidence and skill in using digital technologies) (Agourram et al. 2019; Kholifah et al. 2022).

### 3.3 Data Analysis Techniques and Ethical Considerations

Data analysis involved several steps: descriptive statistics to summarize demographic variables and all main constructs, reliability and validity assessments using Cronbach's alpha (CA) and composite reliability (CR), confirmatory factor analysis (CFA) to examine construct validity, and PLS‐SEM to test the hypothesized relationships between the constructs. The model fit was evaluated using various fit indices such as the chi‐square statistic, RMSEA, CFI, and TLI. Mediation and moderation analysis were conducted using the bootstrapping method to test the mediating effects of URE and SC, and multigroup SEM analysis to examine the moderating effects of TP.

The study adhered to ethical guidelines for research involving human participants. Informed consent was obtained from all participants, and they were assured of the confidentiality and anonymity of their responses. The study was reviewed and approved by the institutional review board (IRB) of the affiliated institution.

The study may face limitations such as self‐report bias and the generalizability of the findings to other populations or contexts. Future research could address these limitations by employing longitudinal designs and exploring additional factors influencing UE.

## 4 Data Analysis

The demographic profile (refer to Table 1) of the respondents reveals several key insights about marathon enthusiasts in China who engage with AI‐driven social media strategies. The majority of respondents are aged 25–34 (67.5%), indicating that young adults are the primary audience for these strategies. This age group is likely to be more tech‐savvy and open to using AI and social media for their marathon training and engagement. Smaller segments are represented by those aged 35–44 (17.5%), 18–24 (3.0%), and 45–54 (6.25%), with minimal representation from those under 18 and over 55. A significant majority of respondents are male (70%), followed by females (27.5%). The low percentages for other genders and those who prefer not to say (both at 1.25%) suggest a gender imbalance typical in marathon participation, reflecting broader societal trends in sports participation and interests in marathon running in China. Most respondents have a higher education background, with 45% holding a bachelor's degree and 12.5% holding a master's degree. This suggests that marathon enthusiasts engaging with AI‐driven social media strategies are generally well‐educated. Lower percentages are seen in those with primary school (1.25%) and secondary school (2.5%) education, indicating a higher level of education is prevalent among the study participants. Regarding running experience, half of the respondents have 3–5 years of running experience (50%), and a substantial number have 1–2 years (22.5%). This distribution indicates that the respondents are relatively experienced runners, likely to benefit from personalized and AI‐driven training tools. Fewer respondents have less than 1 year or more than 10 years of running experience. The largest group of respondents participates in marathons two to three times a year (47.5%), suggesting that regular participation in marathons is common among this cohort. A notable segment participates four to five times a year (25%), while 16.25% participate more than five times a year. Only a small fraction (3.75%) never participate, indicating a high engagement level in marathon activities.

**TABLE 1:** Respondents demographics profile.

Demographic variable	Category	Frequency	(%)
Age	Under 18	3	0.75
	18–24	12	3.0
	25–34	270	67.5
	35–44	70	17.5
	45–54	25	6.25
	55–64	10	2.5
	65 and above	10	2.5
Gender	Male	280	70.0
	Female	110	27.5
	Other	5	1.25
	Prefer not to say	5	1.25
Education level	Primary school	5	1.25
	Secondary school	10	2.5
	High school diploma or equivalent	80	20.0
	Vocational/technical school	30	7.5
	Associate degree	30	7.5
	Bachelor's degree	180	45.0
	Master's degree	50	12.5
	Doctoral degree	15	3.75
Running experience	Less than 1 year	20	5.0
	1–2 years	90	22.5
	3–5 years	200	50.0
	6–10 years	60	15.0
	More than 10 years	30	7.5
Frequency of marathon participation	Never	15	3.75
	Once a year	30	7.5
	Two to three times a year	190	47.5
	Four to five times a year	100	25.0
	More than five times a year	65	16.25
	Never	15	3.75
Frequency of social media use for marathon‐related activities	Rarely (once a month or less)	25	6.25
	Occasionally (two to three times a month)	60	15.0
	Often (once a week)	80	20.0
	Very often (several times a week)	150	37.5
	Daily	70	17.5
Preferred social media platforms for marathon‐related activities	WeChat	320	80.0
	Weibo	190	47.5
	Douyin (TikTok)	140	35.0
	Bilibili	50	12.5
	QQ	90	22.5
	Xiaohongshu (Little Red Book)	60	15.0
	Facebook	20	5.0
	Instagram	25	6.25
	Twitter	10	2.5
	Other	5	1.25
Marathon participation status	Amateur runner	250	62.5
	Semiprofessional runner	90	22.5
	Professional runner	10	2.5
	Coach/trainer	10	2.5
	Event organizer	15	3.75
	Volunteer	10	2.5
	Enthusiast/supporter	15	3.75
Region of residence	North China	60	15.0
	Northeast China	30	7.5
	East China	180	45.0
	South Central China	70	17.5
	Southwest China	30	7.5
	Northwest China	20	5.0
	Other	10	2.5
Employment status	Student	120	30.0
	Employed full‐time	200	50.0
	Employed part‐time	20	5.0
	Self‐employed	25	6.25
	Unemployed	10	2.5
	Retired	10	2.5
	Other	15	3.75

A significant portion of respondents use social media very often (37.5%) or daily (17.5%) for marathon‐related activities. This highlights the critical role of social media in their training and engagement. Those who use it often (once a week) constitute 20%, showing consistent engagement. The least frequent users are those who rarely (6.25%) or occasionally (15%) use social media for such activities. WeChat is the dominant platform, preferred by 80% of respondents, reflecting its popularity and wide reach in China. Weibo (47.5%) and Douyin (TikTok) (35%) are also popular, indicating varied preferences for social media platforms. Other platforms like Bilibili, QQ, Xiaohongshu, Facebook, Instagram, and Twitter have lower usage rates, reflecting diverse but less dominant social media engagement. Most respondents identify as amateur runners (62.5%), followed by semiprofessional runners (22.5%). This suggests that the primary audience consists of nonprofessional but serious runners. Professional runners, coaches/trainers, event organizers, volunteers, and enthusiasts/supporters constitute smaller segments, indicating diverse roles within the marathon community. The majority of respondents reside in East China (45%), followed by South Central China (17.5%) and North China (15%). This distribution may reflect the concentration of marathon events and running culture in these regions. Other regions like Northeast China, Southwest China, and Northwest China have lower representation. Half of the respondents are employed full‐time (50%), and a significant portion are students (30%), suggesting that working professionals and students are the primary users of AI‐driven social media for marathon engagement. Smaller segments are part‐time employees, self‐employed, unemployed, retired, or fall into other categories, showing a diverse employment status among the participants. In summary, the demographic profile of respondents indicates a predominantly young, male, and well‐educated audience with considerable running experience and frequent participation in marathons. They are active social media users, primarily engaging with WeChat, and show varied preferences for other platforms. The data reflect a diverse but skewed distribution typical of snowball sampling, providing valuable insights into the target audience for AI‐driven social media strategies in the marathon context in China.

### 4.1 Measurement Model Statistics

**FIGURE 2:**
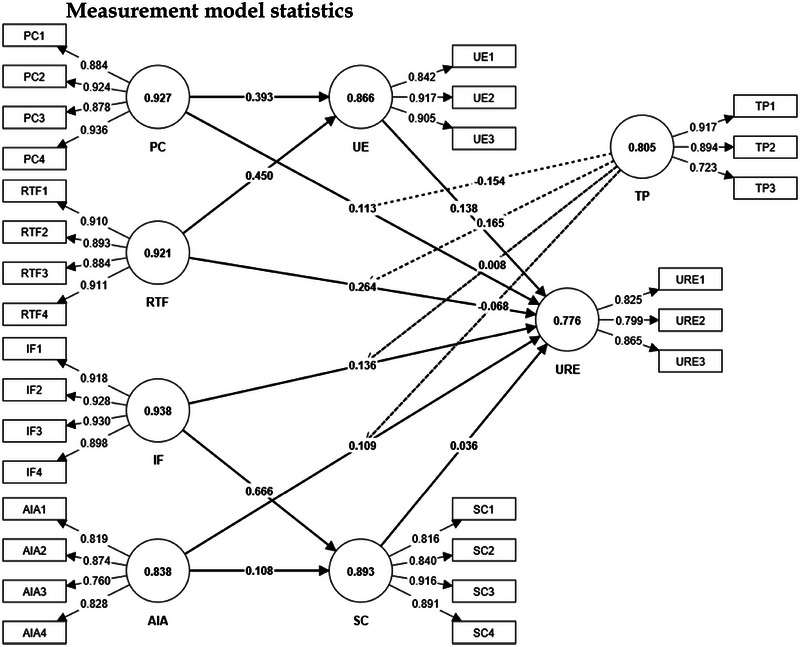
Measurement model.

The measurement model (Figure 2), as presented in Table 2, demonstrates robust psychometric properties for the constructs of AI‐driven analytics (AIA), IF, PC, RTF, SC, TP, UE, and URE. The outer loadings (OLs) for each item exceed 0.70, indicating strong individual item reliability (Shiau et al. 2019). For example, AIA1 (“The AI‐driven analytics provided by the social media platform help me better understand my marathon training progress”) has an OL of 0.819 and a VIF of 1.822, ensuring that multicollinearity is not a concern.

**TABLE 2:** Measurement model statistics.

Construct	Item	Measurement item	OL	VIF	CA	CR (rho_c)	AVE
AIA	AIA1	The AI‐driven analytics provided by the social media platform help me better understand my marathon training progress.	0.819	1.822	0.838	0.892	0.674
	AIA2	I find the AI analytics on the social media platform to be very accurate and useful.	0.874	2.305			
	AIA3	The insights from AI analytics motivate me to improve my marathon performance.	0.760	1.564			
	AIA4	The AI analytics feature on the social media platform is easy to use and understand.	0.828	1.948			
IF	IF1	The live chat feature on the social media platform enhances my engagement with other marathon enthusiasts.	0.918	3.750	0.938	0.956	0.844
	IF2	Q&A sessions with experts on the social media platform are very helpful for my marathon preparation.	0.928	4.190			
	IF3	Interactive features on the social media platform make me feel more connected to the marathon community.	0.930	4.180			
	IF4	I actively participate in interactive features (e.g., polls and live streams) on the social media platform.	0.898	3.031			
PC	PC1	The content recommendations I receive on the social media platform are tailored to my marathon training needs.	0.884	3.174	0.927	0.948	0.820
	PC2	Personalized content on the social media platform helps me stay informed about marathon events and tips.	0.924	4.066			
	PC3	The platform provides content that is relevant to my marathon interests and preferences.	0.878	2.726			
	PC4	I find the personalized content on the social media platform very engaging and useful.	0.936	4.259			
RTF	RTF1	The real‐time feedback on my performance from the social media platform helps me improve my marathon training.	0.910	3.277	0.921	0.944	0.809
	RTF2	I rely on real‐time feedback from the platform to adjust my training routine.	0.893	2.949			
	RTF3	Real‐time performance tracking on the social media platform keeps me motivated.	0.884	2.948			
	RTF4	The immediate feedback I receive on my progress is very beneficial for my marathon preparation.	0.911	3.488			
SC	SC1	I feel a strong sense of community with other marathon enthusiasts on the social media platform.	0.816	3.024	0.893	0.923	0.751
	SC2	The social media platform fosters a supportive environment for marathon runners.	0.840	3.301			
	SC3	I enjoy interacting with other marathon runners on the social media platform.	0.916	3.730			
	SC4	The platform helps me build meaningful connections with fellow marathon enthusiasts.	0.891	3.138			
TP	TP1	I am confident in using digital technologies related to marathon training.	0.917	2.484	0.805	0.885	0.721
	TP2	I find it easy to navigate and use the features on the social media platform.	0.894	2.310			
	TP3	I am proficient in utilizing various technological tools for my marathon training.	0.723	1.403			
UE	UE1	I frequently interact with the content on the social media platform.	0.842	1.833	0.866	0.918	0.790
	UE2	I spend a significant amount of time engaging with marathon‐related content on the platform.	0.917	2.892			
	UE3	I actively participate in discussions and activities on the social media platform.	0.905	2.721			
URE	URE1	My overall experience with the social media platform is very positive.	0.825	1.506	0.776	0.869	0.689
	URE2	I am satisfied with how the platform meets my needs as a marathon enthusiast.	0.799	1.607			
	URE3	The social media platform provides a satisfying user experience for marathon training and engagement.	0.865	1.746			

The constructs also exhibit high internal consistency reliability, with CA values above the 0.70 threshold (Nunnally 1978). For instance, the IF construct has a CA of 0.938, indicating excellent reliability. The CR values are all above 0.70, ensuring that the constructs are reliable (Fornell and Larcker 1981). The average variance extracted (AVE) values exceed 0.50 for all constructs, indicating that each construct explains more than half of the variance of its indicators (Shiau et al. 2019).

Tables 3 and 4 present the discriminant validity results using the heterotrait‐monotrait (HTMT) ratio and the Fornell–Larcker criterion, respectively. The HTMT values for all construct pairs are below the conservative threshold of 0.85, indicating adequate discriminant validity (Henseler et al. 2015). For instance, the HTMT value between AIA and IF is 0.827, which is acceptable. The Fornell–Larcker criterion also supports discriminant validity, with the square root of the AVE for each construct being higher than the correlations with other constructs. For example, the square root of the AVE for AIA is 0.821, which is higher than its correlations with other constructs such as IF (0.736) and PC (0.612).

**TABLE 3:** Discriminant validity (HTMT).

	AIA	IF	PC	RTF	SC	TP	UE	URE	TP x RTF	TP x PC	TP x AIA	TP x IF
AIA												
IF	0.827											
PC	0.690	0.734										
RTF	0.723	0.839	0.779									
SC	0.659	0.780	0.649	0.667								
TP	0.841	0.845	0.780	0.896	0.581							
UE	0.719	0.842	0.797	0.820	0.658	0.803						
URE	0.748	0.791	0.739	0.816	0.653	0.815	0.796					
TP x RTF	0.302	0.437	0.383	0.498	0.207	0.515	0.460	0.389				
TP x PC	0.283	0.358	0.358	0.440	0.189	0.485	0.429	0.409	0.823			
TP x AIA	0.272	0.348	0.281	0.348	0.199	0.447	0.379	0.359	0.782	0.768		
TP x IF	0.333	0.488	0.343	0.481	0.271	0.541	0.469	0.421	0.801	0.835	0.848	

**TABLE 4:** Discriminant validity (FLC).

	AIA	IF	PC	RTF	SC	TP	UE	URE
AIA	0.821							
IF	0.736	0.918						
PC	0.612	0.687	0.906					
RTF	0.638	0.795	0.724	0.900				
SC	0.598	0.746	0.621	0.638	0.867			
TP	0.696	0.747	0.681	0.785	0.537	0.849		
UE	0.618	0.773	0.718	0.734	0.610	0.732	0.889	
URE	0.607	0.682	0.635	0.697	0.565	0.661	0.663	0.830

The demographic and measurement model data together illustrate a comprehensive view of the study context. The young, educated, and predominantly male cohort with substantial running experience suggests a targeted audience likely to benefit from and engage with AI‐driven social media strategies for marathon training and community building. The robust psychometric properties of the constructs ensure the reliability and validity of the measures used in this study, supporting the integrity of the research findings.

### 4.2 Hypothesis Testing and Discussion

**FIGURE 3:**
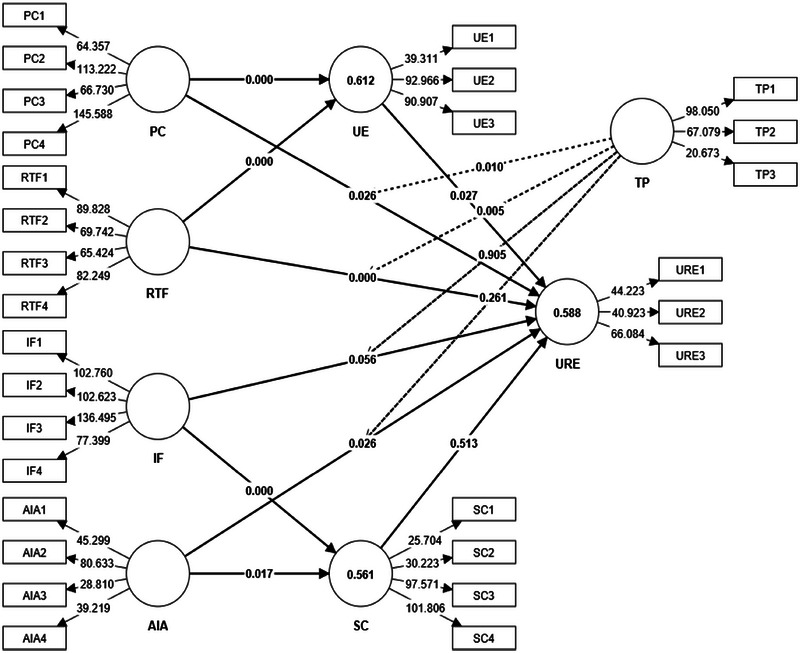
Structural model.

The hypothesis testing results (Figure 3), presented in Tables 5 and 6, provide insights into the relationships between constructs in the study. The model fit statistics demonstrate the adequacy of the structural model, with *R* ^2^ values indicating moderate to substantial explanatory power: SC at 0.561, UE at 0.612, and URE at 0.588. The high predictive relevance (*Q* ^2^ predict) values suggest good predictive ability, and the RMSE and MAE values further support the model's fit.

**TABLE 5:** Model fit statistics.

	*R* ^2^	*R* ^2^ adjusted	*Q* ^2^ predict	RMSE	MAE
SC	0.561	0.560	0.555	0.670	0.472
UE	0.612	0.610	0.607	0.631	0.457
URE	0.588	0.579	0.556	0.670	0.519

**TABLE 6:** Structural model statistics.

Hypothesis path		Original sample	Sample mean	Standard deviation	*T* statistics	*p* values	*f* ^2^	Support
H1a	PC → UE	0.393	0.391	0.052	7.580	0.000	0.189	Yes
H1b	PC → URE	0.113	0.117	0.051	2.225	0.026	0.011	Yes
H2a	IF → SC	0.666	0.666	0.044	15.093	0.000	0.464	Yes
H2b	IF → URE	0.136	0.135	0.071	1.912	0.056	0.008	No
H3a	RTF → UE	0.450	0.450	0.049	9.148	0.000	0.248	Yes
H3b	RTF → URE	0.264	0.258	0.063	4.210	0.000	0.041	Yes
H4a	AIA → SC	0.108	0.110	0.045	2.393	0.017	0.012	Yes
H4b	AIA → URE	0.109	0.110	0.049	2.223	0.026	0.011	Yes
H5	PC → UE → URE	0.054	0.055	0.025	2.194	0.028		Yes
H6	IF → SC → URE	0.024	0.024	0.037	0.648	0.517		No
H7a	TP x PC → URE	−0.154	−0.157	0.060	2.571	0.010	0.022	Yes
H7b	TP x IF → URE	0.008	0.007	0.068	0.119	0.905	0.000	No
H7c	TP x RTF → URE	0.165	0.166	0.059	2.787	0.005	0.021	Yes

The path from PC to UE is significant (*β* = 0.393, *t* = 7.580, *p* < 0.001, *f* ^2^ = 0.189), indicating that PC significantly enhances UE among marathon enthusiasts. This aligns with existing literature emphasizing the importance of PC in digital marketing and UE (Sodiya et al. 2024; Park et al. 2021). PC meets individual preferences, making users feel valued and understood, which enhances their engagement levels (Piduru 2023). The path from PC to URE is also significant (*β* = 0.113, *t* = 2.225, *p* = 0.026, *f* ^2^ = 0.011), though the effect size is small. This suggests that while PC positively impacts URE, other factors like usability, design aesthetics, and overall functionality of the platform also play significant roles (Hassenzahl and Tractinsky 2006).

The impact of IF on SC is highly significant (*β* = 0.666, *t* = 15.093, *p* < 0.001, *f* ^2^ = 0.464), suggesting that IF strongly contribute to fostering an SC among marathon enthusiasts (Mosly 2024). Interactive elements such as live chats, forums, and Q&A sessions can significantly enhance the sense of belonging and community among users (Yang et al. 2022). This strong SC can lead to sustained engagement and loyalty to the platform (Huang et al. 2018). The path from IF to URE is not significant (*β* = 0.136, *t* = 1.912, *p* = 0.056, *f* ^2^ = 0.008), indicating that IF alone do not significantly enhance URE. This suggests that while IF are crucial for building an SC, they do not directly translate into a better URE without other supportive elements like content quality and platform usability (Hassenzahl and Tractinsky 2006; O'brien et al. 2008).

The path from RTF to UE is highly significant (*β* = 0.450, *t* = 9.148, *p* < 0.001, *f* ^2^ = 0.248), suggesting that immediate performance tracking and feedback are crucial for engaging users. RTF provides instant insights into performance, allowing users to adjust their training and see immediate results, which enhances their motivation and engagement (Van Hooren et al. 2019). The influence of RTF on URE is also significant (*β* = 0.264, *t* = 4.210, *p* < 0.001, *f* ^2^ = 0.041), though the effect size is smaller compared to its impact on UE. This indicates that while RTF positively affects URE, its impact is less pronounced, suggesting that URE encompasses broader aspects beyond immediate feedback (Constantiou et al. 2022; Hassenzahl and Tractinsky 2006).

The path from AIA to SC is significant (*β* = 0.108, *t* = 2.393, *p* = 0.017, *f* ^2^ = 0.012), indicating a modest impact of AI analytics on fostering an SC. AIA provide personalized insights and recommendations, helping users feel more connected to their training goals and the broader community of marathon enthusiasts (Mosly 2024). AIA also significantly impact URE (*β* = 0.109, *t* = 2.223, *p* = 0.026, *f* ^2^ = 0.011), though the effect is small. The integration of AI analytics can improve the overall URE by providing personalized recommendations and insights, making the platform more useful and engaging (Nguyen and Sidorova 2017).

The mediation effect of UE between PC and URE is significant (*β* = 0.054, *t* = 2.194, *p* = 0.028), indicating that UE partially mediates this relationship. PC enhances UE, which in turn improves URE, highlighting the role of engagement as a critical intermediary (De Vries et al. 2017). The mediation effect of SC between IF and URE is not supported (*β* = 0.024, *t* = 0.648, *p* = 0.517), suggesting no significant mediation effect (H6). This indicates that while IF are crucial for fostering an SC, this SC does not significantly mediate the relationship between IF and URE (Mosly 2024). Other factors like content relevance and platform usability may play more direct roles in shaping URE.

The moderation effect of TP on the relationship between PC and URE is significant (*β* = −0.154, *t* = 2.571, *p* = 0.010, *f* ^2^ = 0.022), indicating that higher TP diminishes the positive impact of PC on URE (H7a). This suggests that users with higher TP might have higher expectations and thus are less impressed by PC (Hsu and Lin 2023; Venkatesh and Brown 2001). The moderation effect of TP on the relationship between IF and URE is not significant (*β* = 0.008, *t* = 0.119, *p* = 0.905, *f* ^2^ = 0.000), indicating no moderating effect (H7b). This suggests that TP does not influence how IF affect URE, implying that the value of IF is universally recognized and appreciated (X. Zhou et al. 2021). The moderation effect of TP on the relationship between RTF and URE is significant (*β* = 0.165, *t* = 2.787, *p* = 0.005, *f* ^2^ = 0.021), suggesting that higher TP enhances the positive impact of RTF on URE (H7c). Users with higher TP are better able to leverage RTF to improve their URE (Lechermeier et al. 2020; Schneider et al. 2016).

In conclusion, these findings provide a detailed understanding of the interplay between IF, PC, RTF, and TP in shaping UE and URE. While IF are essential for community building, their impact on URE is not mediated by the SC. PC positively impacts URE, but this effect is moderated by TP, with higher proficiency diminishing its impact. Conversely, TP enhances the positive impact of RTF on URE. These insights underscore the importance of considering user skills and expectations when designing AI‐driven social media strategies to effectively engage and satisfy marathon enthusiasts.

## 5 Implications of This Study

### 5.1 Theoretical Implications

This study advances the theoretical understanding of AI‐mediated engagement by deepening the explanatory power of UGT, SDT, and the TAM within digitally embedded fitness communities. Rather than merely applying these frameworks, the study recontextualizes them in light of algorithmic interactivity, where gratification, autonomy, and perceived usefulness arise through dynamic and real‐time AI features.

In relation to UGT, the study demonstrates that users actively seek out PC to meet specific informational and motivational needs. Engagement with AI‐curated content by marathon enthusiasts reflects a targeted media use pattern, where gratification is derived from relevance, timeliness, and personal applicability. However, the nonsignificant mediating role of SC between IF and URE suggests that community‐building tools, in isolation, may not satisfy immediate user motivations unless embedded within broader, goal‐oriented interaction structures.

The findings also extend SDT by showing that RTF contributes meaningfully to UE and URE by reinforcing perceptions of competence and autonomy. AI‐generated feedback enables users to track and adjust their performance in real time, thereby fostering intrinsic motivation. Furthermore, the role of AI analytics in enhancing both relatedness and competence through tailored recommendations illustrates how AI can operationalize key motivational drivers in self‐determined behavior.

TAM is likewise extended through the moderation analysis. TP significantly shapes how users perceive and benefit from AI features. Interestingly, users with higher TP showed diminished responsiveness to PC, suggesting a ceiling effect where basic personalization fails to meet elevated expectations. Conversely, these users benefited more from RTF, affirming TAM's proposition that perceived ease of use enhances usefulness—particularly when features offer meaningful interactivity. The nonsignificant moderation effect for IF underscores their broad appeal and accessibility across skill levels.

By synthesizing UGT, SDT, and TAM, this study offers a theoretically integrated framework to explain how UE emerges from the interplay of motivational, cognitive, and technological factors in AI‐enhanced platforms. This integration moves beyond fragmented applications and contributes to a more cohesive understanding of AI‐enabled URE design.

### 5.2 Practical Implications

This study offers actionable insights for designing AI‐driven social media strategies that effectively engage marathon enthusiasts. The strong influence of PC on UE and experience suggests that platforms should invest in advanced personalization algorithms capable of analyzing individual preferences, training goals, and motivational triggers. Delivering relevant content—such as tailored race updates, training advice, and achievement milestones—can significantly enhance user satisfaction and platform stickiness.

IF remain essential for fostering an SC. Tools such as live chats, forums, and interactive Q&A sessions should be embedded to enable meaningful peer‐to‐peer engagement. These features not only create social cohesion but also sustain long‐term participation by addressing users’ desire for connection and support.

RTF emerges as a critical feature for enhancing user motivation and perceived competence. Integrating wearable technology and performance tracking apps into social media platforms can provide users with immediate insights into their progress. Collaborations with wearable device providers could further enable seamless data flow and contextualized feedback, deepening UE.

AIA, while showing a more modest effect, still contribute positively by offering users personalized performance insights and community alignment cues. Designing AI systems that visualize progress and offer adaptive training suggestions can help users feel more connected to both their personal goals and broader community norms.

Importantly, the study highlights the need for platforms to accommodate varying levels of TP. For more skilled users, offering advanced customization options, granular feedback, and detailed dashboards can elevate the URE. For less proficient users, simplified interfaces, onboarding tutorials, and guided interactions are crucial to ensure usability and access. Additionally, incorporating gamification elements—such as badges, leaderboards, and personalized challenges—can enhance engagement across all proficiency levels.

In sum, AI‐driven engagement strategies should be multilayered, combining personalization, interactivity, and RTF with adaptive design that reflects users' varying digital competencies and motivational profiles.

### 5.3 Contribution to AI Research

This study contributes to the evolving discourse on AI by repositioning it as a participatory and motivational infrastructure in digital environments, rather than a mere vehicle for automation or efficiency. It addresses a key gap in the literature by exploring AI's role in noncommercial, community‐oriented domains—specifically within endurance sport communities—where motivational, behavioral, and social dynamics shape URE.

Theoretically, the integration of UGT, SDT, and TAM presents a holistic model that foregrounds AI as a psychosocial mediator. This synthesis challenges the dominance of utilitarian perspectives in AI adoption research, suggesting that affective and social gratifications are equally vital. The model offers an interpretive lens through which to examine how AI supports needs for autonomy, competence, and relatedness within platform‐based social interaction.

Methodologically, the study advances AI research by employing SEM to capture complex interdependencies between user traits, platform features, and AI functions. This approach elevates the methodological discourse by advocating for contextualized evaluation metrics that go beyond efficiency to include user interpretability, trust calibration, and behavioral responsiveness.

Finally, by situating the research within a digitally mediated sport community, the study extends the applied boundaries of AI research. Unlike prior work focused on e‐commerce or online education, this study addresses the design and impact of AI systems in goal‐oriented, embodied, and socially motivated contexts. It invites future inquiry into how AI can foster long‐term digital well‐being, purpose‐driven engagement, and meaningful community participation across underexplored domains.

## 6 Conclusion and Future Research Directions

This study explored the impact of AI‐driven social media strategies on UE and URE among marathon enthusiasts, using a theoretical framework based on UGT, SDT, and the TAM. The findings confirmed the significant positive effects of PC and RTF on UE and URE. IF were also found to be crucial in fostering an SC, though their direct impact on URE was less pronounced. The moderating role of TP highlighted that users with higher proficiency have different expectations and responses to AI‐driven features, underscoring the need for customizable and user‐friendly platform designs.

The implications for theory and practice are substantial. For theory, this study validates and extends UGT, SDT, and TAM by integrating them into a comprehensive model that explains UE in AI‐driven digital environments. For practice, the study provides actionable insights for designing effective AI‐driven social media strategies, emphasizing the importance of PC, RTF, and IF, while considering the diverse TP of users.

Future research could build on these findings by exploring several avenues. First, longitudinal studies could provide deeper insights into how UE and URE evolve over time with sustained interaction with AI‐driven features. Second, examining the role of cultural differences in user responses to AI‐driven personalization and feedback could offer valuable insights for global platform designs. Third, future studies could investigate the ethical implications of AI‐driven personalization, particularly concerning data privacy and algorithmic biases, to ensure responsible and equitable use of AI technologies. Finally, exploring the impact of emerging technologies such as augmented reality (AR) and virtual reality (VR) on UE and URE in the context of marathon training and events could provide innovative directions for enhancing digital interactions. By addressing these areas, future research can further enrich the understanding and application of AI‐driven strategies in digital platforms.

## Author Contributions

**Wei Cheng**: conceptualization, formal analysis, methodology, project administration, resources, validation, writing–original draft, writing–review and editing. **Yu Tian**: formal analysis, investigation. **Meng Na**: project administration, resources, software, supervision.

Ethics Statement

This study adheres to the ethical standards of Declaration of Helsinki. The study protocol was approved by Human Resource and Ethics Committee of Guangzhou Sport University, China. (Ref. No: GSU20240710).

Consent

Oral consent was obtained from all individuals involved in this study.

## Conflicts of Interest

The authors declare no conflicts of interest.

## Peer Review

The peer review history for this article is available at https://publons.com/publon/10.1002/brb3.70593.

## Data Availability

The data that support the findings of this study are available from the corresponding author upon reasonable request.
